# High diversity and local endemism in Aotearoa New Zealand's groundwater crustacean fauna

**DOI:** 10.1002/ece3.8220

**Published:** 2021-10-24

**Authors:** Graham D. Fenwick, Michelle J. Greenwood, Ian D. Hogg, Stacey J. Meyer

**Affiliations:** ^1^ National Institute of Water and Atmospheric Research Christchurch New Zealand; ^2^ Te Aka Mātuatua/School of Science Te Whare Wānanga o Waikato/University of Waikato Hamilton New Zealand; ^3^ Polar Knowledge Canada Canadian High Arctic Research Station Cambridge Bay Nunavut Canada

**Keywords:** Amphipoda, cytochrome *c* oxidase subunit one (COI), diversity, endemism, groundwater, Isopoda, mitochondrial DNA, stygofauna

## Abstract

We used DNA barcoding to assess the diversity and distribution of New Zealand's groundwater amphipods and isopods (Crustacea) and to determine whether biodiversity and endemism within tectonically active New Zealand are similar to those of more tectonically stable continents. Sixty‐five wells were sampled in seven aquifers across four regions within the North and South islands of New Zealand, and resident invertebrates were morphologically identified and then assessed using sequencing of the mitochondrial DNA cytochrome *c* oxidase subunit one (COI) gene. Invertebrates were found in 54 wells. Of the 228 individual amphipods and isopods found in 36 of the wells, 154 individuals were successfully sequenced for COI (68% success rate) from 25 wells, with at least one well in each aquifer containing sequenced individuals. Of the 45 putative species identified using Barcode Index Numbers (BINs), 30 BINs (78% of all taxa and 83% of amphipods) were previously unrecorded. Substantial morphologically cryptic, species‐level diversity was revealed, particularly within the amphipod Family Paraleptamphopidae. Similarly, one isopod taxon morphologically identified as *Cruregens fontanus* was assigned to five well‐separated BINs based on COI sequences. Endemism appeared high, with all taxa regionally endemic; 87% of species were restricted to one aquifer and more than 50% restricted to one well. Non‐saturated species accumulation curves indicated that, while additional sampling may increase the range of some currently identified taxa, additional range‐restricted taxa are also likely to be discovered. Patterns of diversity and short‐range endemism were similar to those found elsewhere, including locations which are more tectonically stable. The predominance of local endemism within New Zealand's groundwater fauna suggests that land‐use activities and groundwater extraction require careful evaluation to minimize threats to groundwater biodiversity.

## Graphical abstract

We used DNA sequencing of the cytochrome c oxidase subunit one (COI) gene to examine the diversity and distribution of New Zealand's groundwater amphipods and isopods (Crustacea) and to compare diversity and endemism within tectonically active New Zealand relative to more tectonically stable continents. Endemism appeared high, with all taxa regionally endemic; 87% of species were restricted to one aquifer and more than 50% restricted to one well. Patterns of diversity and short‐range endemism were similar to those observed elsewhere, including more tectonically stable locations.

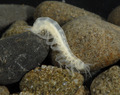

## 1 INTRODUCTION

Groundwater systems are often viewed as lifeless conduits of subsurface water flow (*sensu* Hancock & Boulton, 2008). However, research over the last few decades has identified a rich diversity of groundwater fauna (the stygofauna), which provide important ecosystem services (Griebler et al., 2019). Stygofaunal communities are typically dominated by invertebrates and are characterized by high levels of biodiversity, particularly Crustacea (Danielopol et al., 2000; Gibert & Culver, 2009), and by endemism over small spatial scales (Boulton, 2020; Gibert et al., 2009; Hancock & Boulton, 2008). Logistical difficulties in sampling groundwater ecosystems (Larned, 2012) and the often cryptic morphology of stygofauna (Bradford et al., 2010; Danielopol & Pospisil, 2001; Finston et al., 2007) have meant that biodiversity inventories of subterranean ecosystems are severely lacking in many locations (Ficetola et al., 2019; Gibert & Culver, 2009). Investigating the spatial scales of endemism within groundwater ecosystems is a critical step in understanding the implications of increasing threats, such as water abstraction and contaminant infiltration, as well as the efficacy of different management policies and practices (Boulton, 2020; Mammola et al., 2019).

**FIGURE 1:**
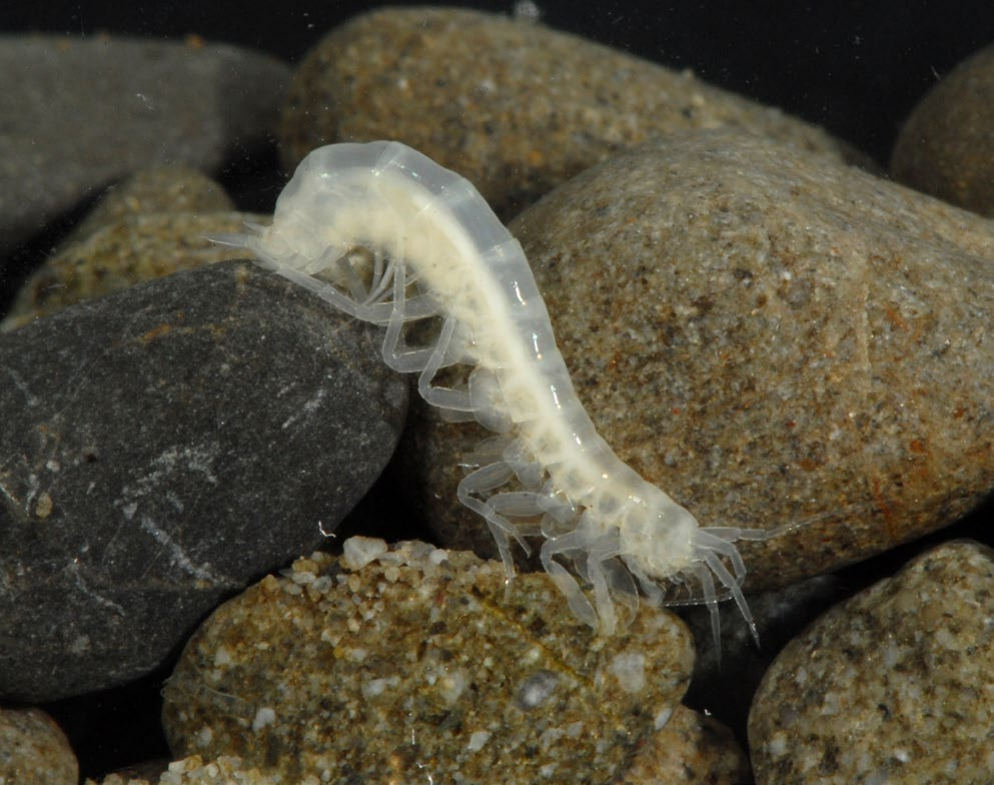
A typical groundwater (stygobitic) crustacean, *Paracrangonyx* sp. (Amphipoda), which shows the lack of pigmentation or eyespots. The head is to the lower right of the photo. Photo credit: N. Boustead

Endemism over relatively small spatial scales appears to be high in most groundwater systems (Danielopol et al., 2003). For example, DNA sequencing of 14 nominal, widespread species indicated more than 50 morphologically cryptic amphipod lineages (Trontelj et al., 2009), most with highly restricted spatial distributions. Forty‐one percent of the stygobitic (obligate groundwater dwellers) species found across six European regions were reported from areas <500 km^2^ (Deharveng et al., 2009) and ranges of <200 km were common (Trontelj et al., 2009). Some taxa were even restricted to a single cave or sampling location (Gibert & Deharveng, 2002). Figure 1 shows a typical stygobitic crustacean, *Paracrangonyx* sp. (Amphipoda), which lacks any pigmentation or eyespots, reflecting its subterranean existence.

For stygobionts, low dispersal capabilities, coupled with geographical isolation over evolutionary time scales, will result in genetic divergences among populations, leading to small‐scale or short‐range endemism (Harvey, 2002; Harvey et al., 2011). Short‐range endemism has been identified in areas that are tectonically stable including Australia (e.g., Hancock & Boulton, 2008) and North America (Culver et al., 2000, 2009) and where physical barriers, such as glacial deposits or catchment shrinkage (due to aridity), subdivide aquifers and isolate populations. New Zealand's active tectonic environment, with relatively rapid uplift, subsidence, erosion, and deposition (Brown, 2001), provides a contrasting setting for stygofaunal evolution compared to that of other continents and could suggest an alternative to the emerging paradigm of short‐range endemism within stygofaunas.

Much of New Zealand's groundwater resides within alluvial aquifers underlying extensive plains comprising relatively young, unconsolidated, and often highly porous matrices, resulting in high hydraulic conductivities and high interstitial water velocities (Close et al., 2002; Pang et al., 1998). This might be expected to facilitate stygofaunal movement within or between aquifers. However, the country's mountainous terrain is also likely to provide physical barriers that would facilitate short‐range endemism. For example, New Zealand's spring and spring‐stream hydrobiid snails include several examples of allopatric, short‐range endemic species (Haase, 2008).

Previous work on New Zealand's stygofauna has largely relied on morphological identifications of taxa, with three families of amphipods and two families of isopods conspicuously present (e.g., Fenwick, 2001; Scarsbrook et al., 2003). This suggests either a smaller number of widespread taxa or a larger number of more restricted, morphologically cryptic taxa. Molecular markers, such as the mitochondrial DNA cytochrome *c* oxidase subunit one gene (COI), are particularly helpful for identifying morphologically conservative taxa, including Crustacea (Costa et al., 2007; Hogg et al., 2006; Watson et al., 2015). For example, molecular studies have revealed that several European stygofaunal species, previously considered widespread within karstic environments of southern and western Europe, actually comprise several morphologically cryptic taxa, each confined to single locations or catchments, with geographic ranges comprising single or multiple localities spanning no more than c. 180 km (e.g., Ferreira et al., 2007; Lefébure et al., 2006, 2007). Similarly, morphologically cryptic, subterranean stygofauna (e.g., amphipods, isopods, and water beetles) inhabiting groundwater calcretes in the arid Yilgarn region of Australia are actually endemic to single calcrete aquifers, with some ranges smaller than a few square kilometers (Cooper et al., 2007).

Here, we assess stygofaunal diversity across New Zealand using COI gene sequences. We focus on amphipod and isopod crustaceans, as they generally dominate the stygofaunal assemblages of shallow alluvial aquifers (Gibert & Deharveng, 2002) and compare stygofaunal diversity and endemism within New Zealand to more tectonically stable continents.

## 2 MATERIALS AND METHODS

### 2.1 Study sites

Sampling locations were stratified hierarchically, across: (1) North and South islands of New Zealand; (2) regions within the South Island; (3) aquifers within regions; and (4) elevation within an aquifer (Figure 1). Sampling was focused on larger alluvial aquifers in four regions along the drier, east coasts of both islands. The largely north–south orientation of New Zealand and its associated mountain ranges, in conjunction with predominantly westerly weather patterns, results in orographic precipitation on the west coast and a drier east coast. Further, we focused on alluvial aquifers to reduce variability in invertebrate communities potentially caused by geographical or hydrogeological differences. These aquifers generally extend from foothills to the coast and have high potentials to store and transport groundwater (Moreau et al., 2019; Tschritter et al., 2017). We collected from the uppermost aquifer at each location. Candidate aquifers were identified using a two‐dimensional aquifer map (Ministry for the Environment, 2015), which was generated using data from White (2001) and updated by Moreau and Bekele (2015). The location of major aquifers corresponds with a more recent map using finer‐scale GIS data (White et al., 2019). Given the unknown quantity of water exchange between aquifers at a small scale, we conservatively assigned wells to the major aquifers identified by both maps and named in Ministry for the Environment (2015). We caution that the “aquifers” identified in this report may comprise two or more smaller aquifers that are variously hydrologically connected.

We assigned a group of wells within the Moutere Valley aquifer to the adjacent Motueka River Terraces aquifer (Figure 1) because our sampling sites were close (440–630 m) and likely hydrologically connected to the Motueka River, which traversed both aquifers. Also, because the Waimea Plains are underlain by multiple major and minor aquifers with some hydrological inter‐connectivity (White, 2001), we assigned our sampling wells in this area to a composite “Waimea Plains” aquifer (Figure 1). There was no established name for the aquifer beneath the Southland wells, so we named this after the nearby Mataura River. Sampling locations within aquifers were restricted to existing wells where sampling equipment could be deployed. Candidate wells were identified with help from local groundwater monitoring agencies (regional and district councils) and had been installed for multiple purposes, including water quality monitoring and research, and for water abstraction.

**FIGURE 2:**
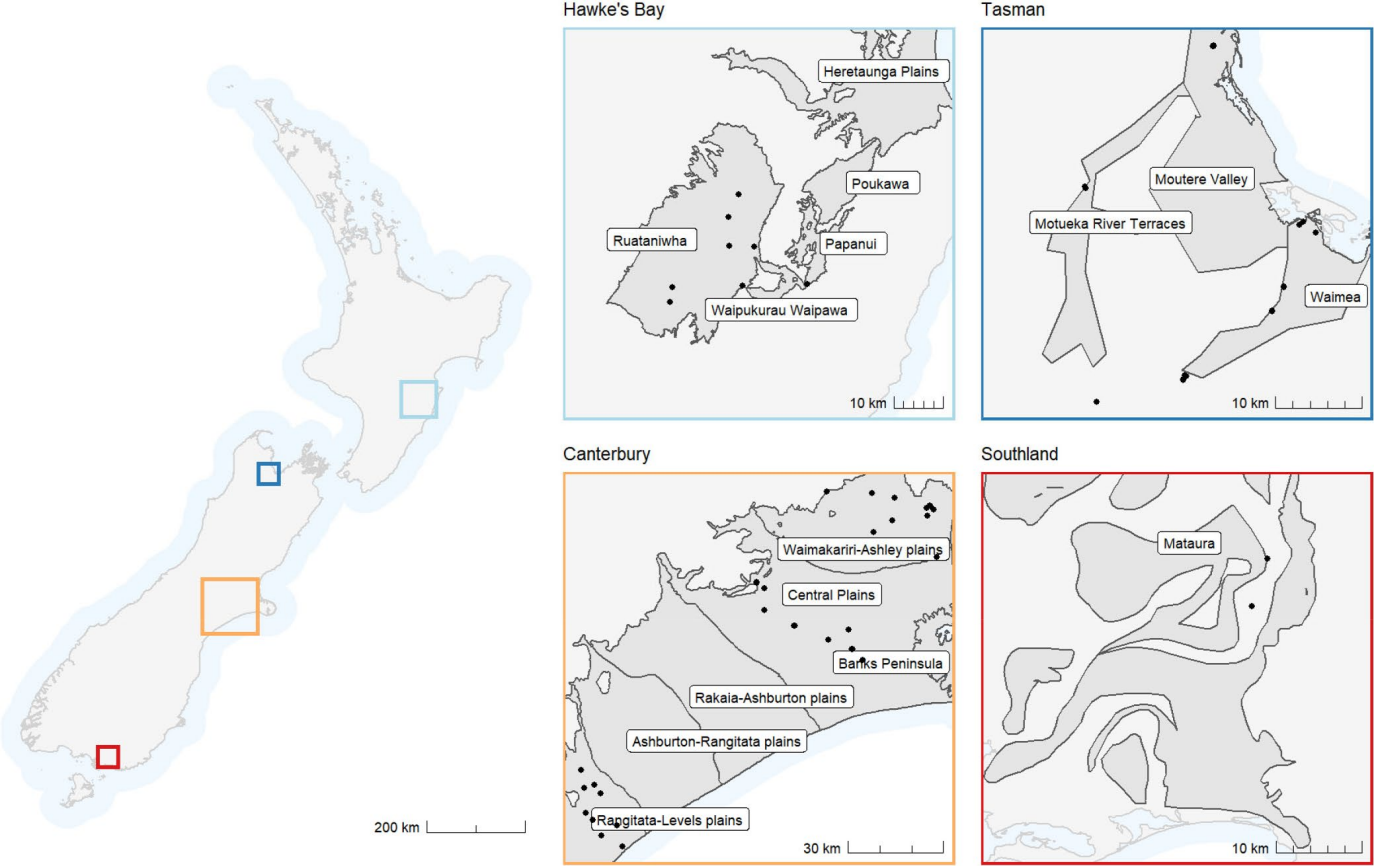
Well locations across four regions of New Zealand with labeled aquifers (from Ministry for the Environment (2015)). Note that wells in the Moutere Valley aquifer were assigned to the Motueka River Terraces aquifer due to a surface water connection nearby (the Motueka River). Waimea is a composite label for multiple aquifers that have some degree of hydrological connectivity and Mataura is an unofficial aquifer name generated for this project. See Appendices 1 and 2 for well details

Sixty‐two wells were sampled once across the four regions and two islands (Figure 2). Due to logistical constraints of finding and accessing suitable wells, the number of wells sampled varied between aquifers. However, at least two wells (and a maximum of 14 wells) were sampled within each aquifer. Where possible, these wells were located across a range of elevations within an aquifer (Appendix 1). We also included invertebrates collected during other sampling excursions from three Canterbury wells (Central Plains aquifer) because they contained specimens that complemented those from the current sampling program. This resulted in a total of 65 wells sampled. We use the term "well" to include both traditional wells (installed by excavation) as well as drilled bore holes. Sampling was undertaken between May 12, 2017, and December 1, 2017, with one well sampled on March 1, 2018.

### 2.2 Invertebrate sampling

We used two main sampling methods to maximize capture rates. Firstly, we pumped 60 or 100 L (depending on well flow rate) of water from the screened (i.e., open to the aquifer via slots or perforations) section of the well through a 200‐µm mesh collecting bag. Neoprene flanges or inflatable packers were used to restrict pumping to the screened section of well. For shallower wells (water table < c. 8 m, 48 wells 77% of wells), we used a Bou‐Rouche pump (Malard et al., 2002). A pneumatic Bennett pump (Bennett Sample Pumps Inc., Amarillo, Texas; pumping rate c. 30 L/min. on average) was used for 11 deeper wells. Secondly, a plankton net (64 or 100 µm mesh, depending on the amount of suspended sediment) with a flexible rim was folded into a weighted bailer, lowered to bottom of the well, bounced to suspend sediment and any associated stygofauna, and retrieved slowly to filter the entire water column. The bailer was then retrieved, and its contents added to the contents of the net haul. This procedure was repeated three times. Contents of repeat net and bailer collections from each well were pooled. Sampling methods were modified for sampling very large and very small wells (casing internal diameters <50 and >500 mm diameter, 5% of wells) where the flanges or packers could not be used. Three samples were also collected coincidentally with other activities (e.g., well conditioning or purging) and employed other field methods and filtered larger volumes of groundwater. In all cases, stygofauna were collected and concentrated using a 200‐µm mesh bag. All samples were preserved in the field with 100% ethanol, chilled, transported to the laboratory, and stored in the dark at −20°C until needed for further processing. All equipment was washed thoroughly after sampling each well and air‐dried between regions to avoid transferring any specimens between wells.

In the laboratory, the contents of each sample were concentrated on a 250‐µm sieve, sorted under a stereomicroscope into separate vials for each recognizable taxon, and stored in the dark at −20°C in 100% ethanol. Amphipods and isopods were identified as far as practicable, based on whole specimen morphology (dissection was avoided to retain material for DNA analyses) using existing literature and guides to the New Zealand stygofauna (Appendix 2).

### 2.3 Physical and chemical parameters

Two 250 ml water samples were collected in acid‐washed bottles from each well between pumping and plankton net sampling to determine dissolved organic carbon content and nutrient concentrations, respectively (nutrients were dissolved reactive phosphorus (DRP), nitrite‐nitrogen (NO_2_‐N), nitrate‐nitrogen (NO_3_‐N), ammoniacal nitrogen (NH_4_‐N), and total dissolved nitrogen (TDN) and total dissolved phosphorus (TDP)). Dissolved oxygen concentration was measured in a five‐liter container of gently pumped well water using a TPS WP‐82 meter (TPS Pty Ltd, Brisbane), and conductivity (µS/cm), temperature, and pH were measured in situ using a TPS WP‐81 meter (TPS Pty Ltd, Brisbane). Well depth and water column depth were measured in situ and information on well diameter and casing material extracted from local council databases.

### 2.4 DNA analyses

Individuals were photographed and loaded into single wells on 96‐well microplates for processing at the Canadian Centre for DNA Barcoding (CCDB). Total DNA was extracted from specimens using a glass fiber plate method (Ivanova et al., 2006, 2007). Following DNA extraction, residual cuticular material for each specimen was deposited with the NIWA (National Institute of Water and Atmospheric Research Ltd, Wellington) Invertebrate Collection (NIC) as museum vouchers available for morphological study.

Polymerase chain reaction (PCR) amplification of the mitochondrial cytochrome *c* oxidase subunit I (COI) gene region used the primer pairs LepF1 and LepR1 (Hebert et al., 2004) and LCO490 and HCO2198 (Folmer et al., 1994) according to CCDB standard protocols (Ivanova & Grainger, 2007). Successfully amplified products progressed to cycle sequencing using BigDye^™^ v3.1 terminator chemistry (Applied Biosystems^™^). Products were then cleaned using a semi‐automated AutoDTR^™^ method (EdgeBio^®^) before being sequenced in forward and reverse directions on an ABI 3730xl DNA Analyzer (Applied Biosystems^™^) using the same primers used for PCR amplification.

Specimen images, collection data, raw trace files, and edited sequences were all uploaded to and are available on the Barcode of Life Datasystems (BOLD) database (Ratnasingham & Hebert, 2007) (http://dx.doi.org/10.5883/DS‐GDWMS) and cross‐referenced to GenBank (accession numbers OK072722‐OK072875). Barcode Index Numbers (BINs; Ratnasingham & Hebert, 2013) assigned by BOLD were used to delineate putative species based on the sequence data (Milton et al., 2013).

### 2.5 Data processing and statistical analyses

The amphipod sequences were aligned in Geneious Prime 2020.0.4 (Kearse et al., 2012, https://www.geneious.com) using MUSCLE (Edgar, 2004) and trimmed to 462 bp. A Maximum‐likelihood (ML) phylogenetic tree was generated in MEGA7, (Kumar et al., 2016) with GTR+G+I used as the model of evolution and 1000 bootstrap replications. Similarly, the isopod sequences were aligned and trimmed to 488 bp and a ML phylogenetic tree was generated with TN93+G+I used as the model of evolution and 1000 bootstrap replications. The final trees were visualized in FigTree v1.4.4 (http://tree.bio.ed.ac.uk/software/figtree/).

We calculated species accumulation curves using the abundance of BINs within each well to investigate how sampling effort (number of wells) affected the diversity of BINs both at the national and at regional scales. If most species within an area are collected, an asymptote in cumulative species richness is expected as sample number increases. The mean, median, and variance (quartiles and range) of species richness estimates for each additional well (1‐n wells with genetic sequences) were calculated from 100 permutations with wells added in random order using the package Vegan in the statistical program R (R Development Core Team, 2017).

Spearman rank correlation was used to assess whether the diversity of BINs detected within a well was positively correlated with the number of sequenced individuals. To investigate potential range restriction and, thus, endemism at different scales, the spatial occurrence of individual BINs between aquifers, regions, and islands was also assessed. Separate one‐way analyses of variance (ANOVA) were used to assess whether wells that contained specimens with successful COI sequences differed in physical and chemical parameters from wells that either contained no stygofauna or from which successful sequences were not generated. These parameters included well depth, spot measurements of water temperature, conductivity and pH, and nutrient concentrations (DRP, TDN, TDP, nitrate‐N, nitrite‐N, ammoniacal‐N). For each assigned aquifer, the diversity of amphipods per 1000 km^2^ of surface catchment area (from Booker & Whitehead, 2017) and aquifer area (from Ministry for the Environment, 2015) was calculated.

## 3 RESULTS

**FIGURE 3:**
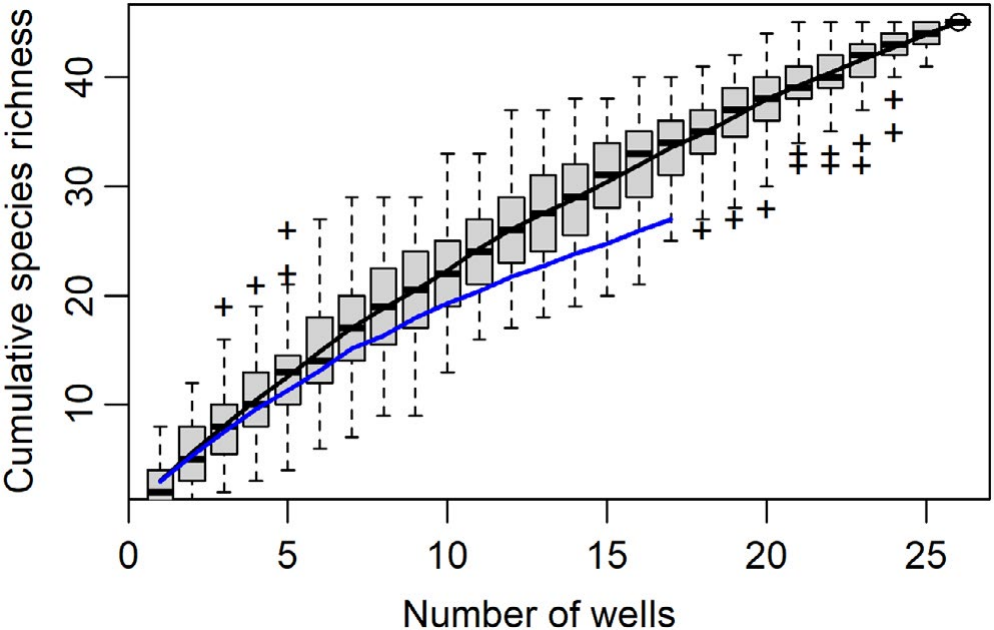
Species accumulation curves showing average cumulative taxa richness (combined amphipod and isopod BINs) against the number of wells sampled for wells within Canterbury (blue line, *n* = 17) and for all wells sampled (black line, *n* = 25 wells with BINs). Boxplots show the variance of species richness estimates from 100 permutations with wells added in random order for all wells (black bar =median, box indicates first to third quartile, with outliers indicated by a cross)

**FIGURE 4:**
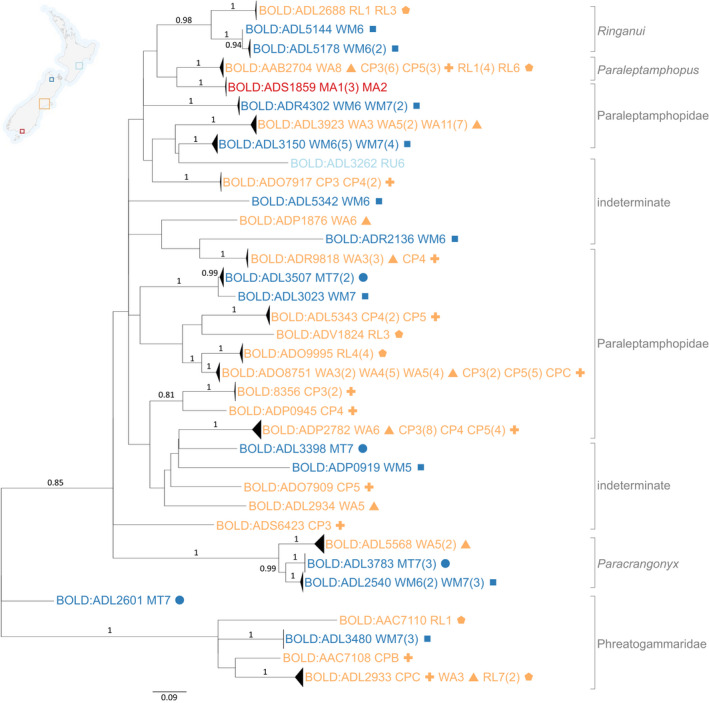
Maximum‐likelihood tree (MEGA7; GTR+G+I) based on aligned and trimmed COI sequences (462 bp) of groundwater amphipods. Supporting bootstrap values >0.7 (1000 replicates) are provided. Labels are color‐coded according to region/aquifer and symbols are used to differentiate sites within the regions: Hawke's Bay, light blue (RU, Ruataniwha); Tasman, dark blue (MT, Motueka, circle (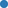); WM, Waimea, square (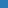)); Canterbury, orange (WA, Waimakariri‐Ashley, triangle (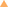); CP, Central Plains, plus sign (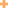); RL, Rangitata Levels, pentagon (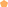)), Southland, red (MA, Mataura). Tentative morphological identifications are provided at the far right of the figure. Barcode of Life Datasystems (BOLD) Barcode Index Numbers (BINs) are shown at the branch tips. Different BINs represent putative species‐level differences. Aquifer and well codes as well as number of individuals (in parentheses) are shown following each BIN. For further details on well locations, see Figure 2 and Appendix 3

Of the 65 wells sampled, 54 (83%) contained stygofauna with amphipods found in 34 wells (52%) and isopods in 15 wells (23%) (Table 1). All amphipod and isopod specimens were considered to be stygobionts as they lacked body pigments or pigmented eyes (c.f., Marmonier et al., 1993). Other taxa that were found in more than five wells included cyclopoid and harpacticoid copepods, Syncarida, Ostracoda, Acarina, Annelida, Nematoda, and Gastropoda. From the wells containing amphipods and isopods, 186 amphipods and 42 isopods were collected and processed for their COI sequences, with successful sequences obtained from 154 individuals (68% overall success rate). Morphological identification to family or occasionally genus was possible for most specimens. Mounting and dissection of specimens for morphological assessment were largely precluded as many of the specimens were very small (e.g., adult, brooding amphipods <2 mm long) or their often damaged condition meant that any available tissue was required for DNA extraction. However, subsequent re‐examination of morphologies following COI sequencing (based on their clustering within trees; Figures 3 and 4), allowed us to allocate further specimens to established families or genera.

**TABLE 1:** Numbers of wells within the four New Zealand regions and seven aquifers that were sampled for stygofauna, in which stygofauna (all stygofauna, amphipods, and isopods) were collected, and from which COI gene sequences were successfully obtained (amphipods, isopods, and combined amphipods and isopods)

Island	Region	Aquifer(s)	Sampled	Taxa present			Successful COI sequences		
				All stygofauna	Amphipoda	Isopoda	Amphipoda	Isopoda	Total
North	*Hawke's Bay (9 wells)*								
		Ruataniwha	9	4	2	1	1	0	1
South	*Tasman (19 wells)*								
		Motueka R Terraces	7	7	2	1	1	1	2
		Waimea Plains*	12	10	4	3	3	2	3
	*Canterbury (35 wells)*								
		Waimakariri‐Ashley Plains	12	9	7	3	6	2	6
		Central Plains	14	13	9	5	5	4	6
		Rangitata Levels Plains	9	9	8	2	5	2	5
	*Southland (2 wells)*								
		Mataura**	2	2	2	0	2	0	2
Total			65	54	34	15	23	11	25

Note

Aquifer names are modified from Ministry for the Environment (2015). * refers to complex of multiple aquifers; ** “Mataura” is the name of a nearby river and not an official aquifer name.

Of the 228 individual crustaceans collected, successful sequences were obtained from 129 amphipods (69% success) and 25 isopods (60% success). These sequences were obtained from a total of 25 wells, with amphipods and isopods successfully sequenced from 23 and 11 wells, respectively (Table 1). Seventeen of the wells were in Canterbury, with 5–6 wells in each of the aquifers (Table 1). Although sequences from the Canterbury region were over‐represented, most aquifers, apart from the Ruataniwha aquifer in Hawke's Bay, contained at least two wells where sequences were obtained (Table 1). Individually, amphipod taxa were successfully sequenced from at least one well in each aquifer. However, isopod sequences were available only for the Canterbury and Tasman aquifers (Table 1).

The 154 COI sequences were assigned to 45 BINs, comprised of nine isopod BINs and 36 amphipod BINs (Table 2). The number of specimens available for sequencing and success of sequencing differed between regions, catchments, and wells within catchments (Table 2). The number of sequenced individuals per well ranged from one to 23 (median three), with six wells having only one sequenced individual. The number of BINs per well ranged from one to eight (median two), with 11 wells containing a single BIN. Wells with more sequenced individuals had greater BIN diversity (*r* = 0.84, *p* < .001). Species accumulation curves (based on BINs) were unsaturated both for the Canterbury region and for all wells combined (Figure 3).

**TABLE 2:** Numbers of successfully sequenced individuals and BINs for amphipods and isopods within each region (bolded values) and aquifer

Region	Aquifer	Amphipoda		Isopoda		TOTALS	
		Seq.	BINs	Seq.	BINs	Seq.	BINs
Hawke's Bay	Ruataniwha	**1**	**1**	**0**	**0**	**1**	**1**
Tasman		**34**	**14**	**6**	**2**	**40**	**16**
	Motueka R Terraces	7	4	1	1	8	5
	Waimea Plains	27	10	5	1	32	11
Canterbury		**90**	**20**	**19**	**7**	**109**	**27**
	Waimakariri‐ Ashley	31	9 (33%)	3	2 (80%)	34	12 (42%)
	Central Plains	44	12 (58%)	13	5 (50%)	57	17 (64%)
	Rangitata Levels	15	6 (67%)	3	1	18	7 (71%)
Southland	Mataura	**4**	**1**	**–**		**4**	**1**
TOTALS		129	36 (83%)	25	9	154	45

Note

The en dash indicates no specimens analyzed. Amphipoda and region BINs (bolded) exclude occurrences in more than one aquifer. Numbers in parentheses within the BIN columns indicate the percentage of amphipod, isopod, and total BINs unique to each aquifer. If no number is included, then all BINS are unique (100%).

Thirty‐five amphipod BINs (78% of all taxa and 83% of amphipods) were new records on BOLD. Of these, only three could be attributed to known morphologically described genera. The endemic amphipod genus *Ringanui* was assigned to three BINs (Figure 3). The Canterbury/Rangitata Levels BIN (ADL2688, Figure 3) probably comprised one of the two described species (reported range Waimakariri‐Ashley to Rangitata Levels aquifers and Temuka; Fenwick, 2006), whereas two other BINs (ADL5144, ADL5178) are probably undescribed species endemic to the Waimea aquifer. Substantial morphologically cryptic diversity was identified at the family level. The Family Paraleptamphopidae includes three described genera, two of which are hypogean, whereas our analysis found 27 BINs representing several potential genera within a large paraleptamphopid clade (Figure 3).

**FIGURE 5:**
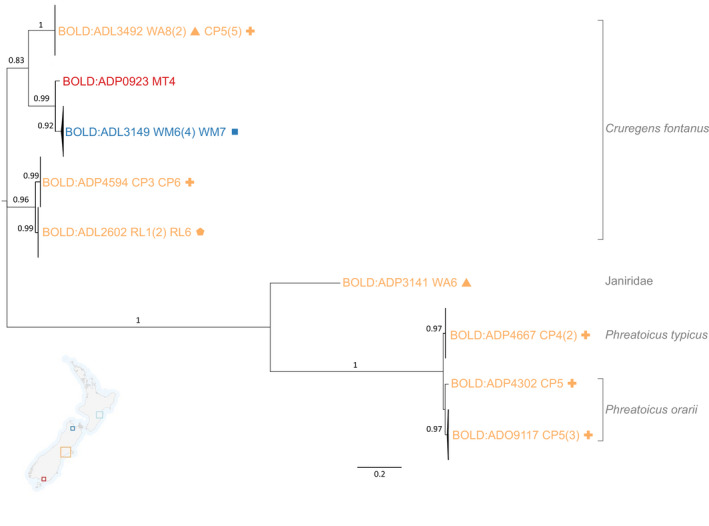
Maximum‐likelihood tree (MEGA7; TN93+G+I) based on aligned and trimmed COI sequences (488 bp) of groundwater isopods. Supporting bootstrap values >0.7 (1000 replicates) are provided. Labels are color‐coded according to region/aquifer and symbols are used to differentiate sites within the regions: Tasman, dark blue (WM, Waimea, square (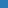)); Canterbury, orange (WA, Waimakariri‐Ashley, triangle (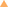); CP, Central Plains, plus sign (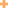); RL, Rangitata Levels, pentagon (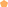)), Southland, red (MA, Mataura). Tentative morphological identifications are provided at the far right of the figure. Barcode of Life Datasystems (BOLD) Barcode Index Numbers (BINs) are shown at the branch tips. Different BINs represent putative species‐level differences. Aquifer and well codes as well as number of individuals (in parentheses) are shown following each BIN. For further details on well locations, see Figure 2 and Appendix 3

Cryptic diversity was also apparent within the Isopoda. Eight specimens that were originally morphologically identified as *Cruregens fontanus* were assigned to five well‐separated BINs (>92% support, Figure 5), one each in Motueka and Waimea aquifers (BINs ADP0923, 3149), one shared between aquifers within the Canterbury region (ADL3492) and two appear to be single‐aquifer endemics (ADL2602, ADP4594) within the Central Plains aquifer (Figure 5). Similarly, three BINs of the phreatoicid isopods were distinguished (>91% support) from specimens initially identified as *Phreatoicus typicus* and *P*. *orarii* (Figure 5).

Each of the 45 genetically distinct isopod and amphipod BINs was restricted to one region; 27 BINs were unique to Canterbury, 16 to Tasman, one to Hawke's Bay, and one to Southland (Table 2). Each BIN from the Tasman region was found in a single aquifer only: 11 BINs were unique to the Waimea aquifers and five to the Motueka River terrace aquifer. In Canterbury, 11 BINs were specific to the Central Plains aquifer, five to the Waimakariri‐Ashley, and five to the Rangitata Levels aquifer (Table 2). Six BINs (one isopod and five amphipods) were found in both the Waimakariri‐Ashley and Central Plains aquifers, and two of these occurred across the three Canterbury aquifers. Many amphipod BINs (86%) were apparently endemic to single aquifers (Figure 4, Table 2). However, in more intensively collected aquifers (Waimakariri‐Ashley and Central Plains), single‐aquifer endemics only comprised 46% and 33% of BINs, respectively. These aquifers shared five amphipod BINs, two of which spanned all Canterbury aquifers sampled (Ashley to Rangitata Levels, c. 100 km; Figure 4). Most isopod BINs (89%) were also from single aquifers while specimens assigned to one BIN (ADL3492) were collected in two aquifers (Central Plains, six specimens; Waimakariri‐Ashley, two specimens) (Figure 4).

Of the 39 BINs (87%) restricted to individual aquifers, 29 (64% of all BINs) were found at one well within the aquifer, and nine (20%) BINs confined to a single aquifer were found in two wells. Three BINs occurred at three wells within an aquifer.

Increasing spatial separation was commonly correlated with greater genetic variability. For example, specimens of the Family Paracrangonyctidae from the Motueka aquifer (ADL3783) were genetically distinct from Waimea specimens (ADL2540) (Figure 4), and those from Canterbury's Waimakariri‐Ashley system (ADL5568) were even more genetically divergent, reflecting the much greater geographic distance of the Waimakariri‐Ashley system from the two Tasman aquifers (c. 150 km cf. <1 km between adjacent headwater tributaries).

We found very high amphipod diversity in four aquifers within two of the regions studied. Comparisons based on estimated numbers of BINs per 1000 km^2^ of aquifer area and catchment area (Table 3) reveal very low relative richness in the two largest, but poorly sampled aquifers (<1.5; Tukituki, Mataura), intermediate richness (3.9 BINs/1000 km^2^) in the most intensively sampled Selwyn/Central Plains aquifer, and high richness in the three more intensively sampled aquifers (>6.0 BINs/1000 km^2^). Highest estimated richness (>80 BINs/1000 km^2^) was within the Waimea aquifer.

**TABLE 3:** Total number of amphipod BINs (putative species) from each catchment and aquifer sequenced during present investigation, total land area for each sampled catchment and aquifer (from Booker & Whitehead, 2017), and estimated amphipod species richness per 1000 km^2^ each catchment and aquifer. Moteuka = Moutere Valley aquifer + Motueka River Terraces

Catchment/aquifer	Amphipod species/BINs	Catchment		Aquifer	
		Total area km^2^	Amphipod species/1000 km^2^	Total area km^2^	Amphipod species/1000 km^2^
Tukituki/Ruataniwha	1	2500	0.4	806.61	1.2
Motueka	4	2056	2.0	387.75	10.3
Waimea	10	770	13.0	120.25	83.1
Ashley/ Waimakariri‐Ashley Plains	9	4758	1.9	1358.08	6.6
Selwyn/ Central Plains	12	974	12.3	3045.59	3.9
Orari/ Rangitata Levels Plains	6	715	8.4	971.28	6.2
Mataura	1	5356	0.2	4337.99	0.2

### 3.1 Physical and chemical parameters

The sixty‐five sampled wells varied in physical size (depth range: 2.7–39 m, diameter range: 50–1200 mm), chemical parameters (e.g., conductivity range 1.2–1014 μS), and nutrient status (e.g., NO_3_‐N range 1.0–11,000 mg/m^3^, Appendix 1).

The water in wells from which specimens with successful genetic sequences were collected was cooler (median spot water temperature 12.9°C) than wells where either taxa were not collected or genetic sequencing failed (median water temperature 13.5°C, ANOVA: *F*_1,53_ = 7.1, *p* = .03). Likewise, conductivity was lower in wells that yielded successful sequences (median 120.9 μS) than those that did not (median 167.2 μS, ANOVA *F*_1,53_ = 4.3, *p* = .04, Appendix 3). There were no differences in well depth or nutrient concentrations between wells that had specimens resulting in successful COI sequences compared with wells where no successful sequences were obtained, or from which no amphipods or isopods were collected. Seventy percent (n = 18) of the wells from which successful COI sequences for amphipods and isopods were processed had steel casings, while six wells had PVC casings and two were undetermined. No amphipod or isopod specimens were collected from the larger (>400 mm diameter) concrete wells. The elevation of wells with COI sequences ranged from 4 to 216 m a.s.l. (median 82.5 m) and well depth ranged from 2.7 to 26 m (median 9.7 m; Appendix 3).

## 4 DISCUSSION

Of the 45 putative species (BINs) identified from the COI sequences, 78% were previously unrecorded on BOLD. Of these, only three could be attributed to established genera, indicating that current knowledge of New Zealand's stygofaunal diversity is extremely low. Morphologically cryptic taxa were common, as has been found in other genetic studies of groundwater taxa (e.g., Delić et al., 2017; Eme et al., 2018). For example, one currently recognized isopod species (*Cruregens fontanus*) was assigned to five well‐separated BINs, and over 20 species were found within the Family Paraleptamphopidae, particularly within the genus *Paraleptamphopus*. Six specimens of phreatoicid isopods also showed cryptic diversity. Specifically, three BINs were found in the vicinity of the Central Plains aquifer, whereas Chilton (1894), and Wilson and Fenwick (1999) previously reported a single species within the Central Plains and Waimakariri‐Ashley aquifers. The few examples of taxa found in more than one aquifer were possibly stygophilic, migrating between aquifers via permanent and/or intermittent surface water connections.

Endemism appeared high, with all species found in only one region, 87% attributed to single aquifers and more than 50% recovered only from single wells. However, we caution that the actual levels of local endemism (e.g., well, aquifer) are likely to be somewhat lower, as our sampling within individual wells and aquifers was not comprehensive (we obtained successful sequences for isopods and amphipods from 38% (*n* = 25) of the 65 wells sampled). Due to their subterranean nature, the sampling of groundwater ecosystems is inherently challenging (Hancock & Boulton, 2009; Korbel et al., 2017). Specifically, collection is often restricted to pre‐existing wells that are predominantly located in areas of human activities. Likewise, it can be difficult to effectively deploy sampling equipment within a well and samples may not adequately represent invertebrate biodiversity within the surrounding aquifer (Ficetola et al., 2019; Larned, 2012), particularly when sampling is only possible on a single occasion. For example, in the Pilbara region of Australia, multiple sampling methods and multiple visits were required to capture most of the species present within a given well (Eberhard et al., 2009).

In our study, only aquifers that were better sampled (i.e., more wells and more sequenced individuals), such as the Canterbury aquifers, yielded taxa that were not aquifer specific. This implies that further sampling is likely to increase the geographic range of some of the apparent single‐aquifer endemic taxa. However, between 42% and 71% of taxa in our most intensively sampled region (Canterbury) were aquifer‐specific indicating that additional sampling would also reveal additional species, many of which are likely to be range‐restricted. In the United States, sampling over nearly 40 years after an early survey of cave fauna led to a <20% decline in frequency of reported county‐specific endemism, while the absolute number of endemic species increased nearly threefold (Culver et al., 2000). While we are unable to definitively identify aquifer‐specific taxa based on our study, other studies of groundwater stygofauna indicate that aquifer‐specific endemism is likely to be common (e.g., Culver & Sket, 2000; Ferreira et al., 2007; Gibert et al., 2009; Bradford et al., 2010; Murphy et al., 2013).

We identified 36 putative stygofaunal amphipod species, 20 in Canterbury region aquifers, and at least 12 within the Central Plains aquifer (Appendix 4). Assuming similar aquifer‐specific endemism across New Zealand's 15 regions and the 220 larger, named aquifers (Moreau et al. (2019), simple extrapolation suggests there could be as many as 300 to 2600 species of groundwater amphipods across New Zealand. Future collecting, particularly repeated sampling and more accurate diversity extrapolation techniques (Eberhard et al., 2009), would assist in more accurately quantifying the diversity of New Zealand groundwater stygofauna, as simple extrapolation techniques are likely to be somewhat limited (Culver et al., 2012). However, the likely diversity identified here is within the range of total stygofaunal diversity in other regions across the world. The described (named) groundwater biodiversity of all aquatic stygofauna in France, after 200 years of study, stands at 380 species, although this is likely to be an under‐estimate due to incomplete sampling (Ferreira et al., 2007) and the presence of several morphologically cryptic species (e.g., Wattier et al., 2020; Westram et al., 2011). In the United States, 300 species of cave‐dwelling aquatic groundwater species are known (Culver et al., 2000). Extrapolating sampling effort and species caught suggests that the Pilbara region of Western Australia may contain 500–550 species (Eberhard et al., 2009) and 21 species of amphipods were present in Australia's smaller Yilgarn region (Cooper et al., 2007).

We found high richness of stygofaunal amphipods in three of the more intensively sampled aquifers (>6.0 BINs/1000 km^2^). We also estimated extremely high stygofaunal amphipod richness (>80 BINs/1000 km^2^) within the Waimea aquifer, probably resulting from its interconnectedness with two other aquifers and the extremely high hydraulic transmissivity (20,000 m^2^/day) reported for parts of this aquifer, including adjacent to the rivers (White & Rosen, 2001). These amphipod richness values are similar to the highest reported for total stygofaunas elsewhere: 6.6 total stygofaunal species/1000 km^2^ for karst in the Balkan Peninsula (Deharveng et al., 2009; Gibert et al., 2009), although those measures of richness preceded DNA investigations which revealed substantial cryptic stygofaunal diversity. The New Zealand stygofaunal amphipod richness is twice that reported for the total stygofauna in the Pilbara region of Australia (or 3.1 species/1000 km^2^) and much greater than that reported for amphipods from Australia's Yilgarn region (0.05 species/1000 km^2^; Cooper et al., 2007). The higher richness found in these New Zealand aquifers is more remarkable because it does not include other taxa such as isopods, copepods, ostracods, syncarids, platyhelminths, and oligochaetes known from these New Zealand aquifers (e.g., Fenwick, 2000; Larned et al., 2014; Scarsbrook & Fenwick, 2003; Scarsbrook et al., 2003).

As with stygofaunas elsewhere, many of described and new species of amphipods and isopods inhabiting New Zealand's alluvial groundwater likely have restricted geographic distributions. The number, size, and complexity of New Zealand's aquifer systems, hydrologically separated by extensive hills and mountains, are probable reasons for the country's high stygofaunal diversity. This appears true even where headwater tributaries almost join (e.g., Motueka and Waimea catchments). Stygofaunal populations also appear genetically isolated between aquifers within the relatively homogeneous landscape of Canterbury Plains, where there are no obvious geohydrological barriers. However, the complexity of the plains’ subsurface geology and hydrogeology (Bradshaw & Soons, 2008; Davey, 2006) may hydrologically separate individual aquifers, leading to genetic isolation and at least some, short‐range, endemism.

Climatic events appear to be the main factor in changing hydrological connectivity and genetic isolation for tectonically stable continents like Europe, North America, Australia, and Africa (Collins et al., 2019; King & Leys, 2014; Lefébure et al., 2006; Witt et al., 2006). Both glaciations and aridity are strongly implicated in creating hydrological barriers that isolated populations of stygofauna, leading to genetic divergence and speciation. These two types of climatic events are also likely to be important drivers of hydrological and genetic isolation of aquifers within New Zealand's alluvial plain systems.

Tectonic events may also have a role via lateral and/or vertical displacement creating barriers and/or changing groundwater flow directions (Trontelj et al., 2009; Craw & Waters, 2007). For example, most (if not all) of New Zealand's larger plain systems are fragmented with recent and historic faults, including the Pacific–Australian tectonic plate boundary. The effect of active faulting (e.g., the Greendale Fault responsible for the 2011 Christchurch eartquake), on dispersal and gene flow is unknown, although it may create physical barriers within an aquifer. Shearing and shaking could consolidate alluvium, reduce interstitial pore spaces and hydrological connectivity, or uplift may misalign strata to subdivide an aquifer (Cox et al., 2012; Rutter et al., 2016). Alternatively, tectonic activity may breach existing hydrological barriers between adjacent aquifers (e.g., bedrock fractures through ranges or breaks in confining layers may create new hydrological connections) and facilitate gene flow.

### 4.1 Summary

There have been repeated calls for accelerated scientific work to identify groundwater biodiversity, which is threatened with extinction before being discovered, identified, and ideally assigned a conservation status and protected (Gladstone et al., 2021; Mammola et al., 2019). Like most countries, knowledge of groundwater fauna is exceptionally poor in New Zealand. Our results support common findings of high biodiversity and short‐range endemism in groundwater faunas internationally (e.g., Boulton, 2020; Gladstone et al., 2021) and likewise for the use of genetic data in identifying morphologically cryptic species, which are common in groundwaters (Boulton, 2020; Delic et al., 2017; Eme et al., 2018; Gladstone et al., 2021). By contributing to knowledge of the biodiversity and spatial distributions of groundwater taxa, we hope to help address some of the knowledge gaps inhibiting conservation of groundwater biodiversity (e.g., Boulton, 2020; Mammola et al., 2019, 2020).

## CONFLICT OF INTEREST

The authors declare no conflicting interests.

## AUTHOR CONTRIBUTION

**Graham D. Fenwick:** Conceptualization (lead); Funding acquisition (lead); Investigation (equal); Methodology (equal); Project administration (equal); Resources (equal); Supervision (equal); Validation (equal); Visualization (equal); Writing‐original draft (lead). **Michelle J. Greenwood:** Formal analysis (equal); Investigation (equal); Methodology (equal); Project administration (equal); Validation (equal); Visualization (equal); Writing‐review & editing (equal). **Ian D. Hogg:** Data curation (equal); Formal analysis (equal); Investigation (equal); Methodology (equal); Project administration (equal); Resources (equal); Supervision (equal); Validation (equal); Writing‐review & editing (lead). **Stacey J. Meyer:** Data curation (equal); Formal analysis (equal); Investigation (equal); Methodology (equal); Visualization (equal); Writing‐review & editing (equal).

## Data Availability

Residual cuticular material after DNA extraction for each specimen was deposited with the NIWA (National Institute of Water and Atmospheric Research Ltd, Wellington) Invertebrate Collection (NIC) as museum vouchers available for morphological study. Specimen images, collection data, raw trace files, and edited sequences were all uploaded to and are available on the Barcode of Life Datasystems (BOLD) database (https://doi.org/10.5883/DS‐GDWMS) and cross‐referenced to GenBank (accession numbers OK072722‐OK072875). All data associated with the paper are also available from the NZ Biological Heritage National Science Challenge data repository at https://doi.org/10.34721/qk3f‐6y64.

## APPENDIX 1

Summary environmental characteristics for the 65 sampled wells. The number of sites in each category is listed in the median column for categorical variables. Some wells were missing environmental data owing to logistical sampling constraints and equipment failure

**Table:**

Parameter	Description	No. of wells missing data (%)	Median	Range
Well depth (m)	Depth of well in meters	0	9.2	2.7–38.7
Water column depth (m)	Depth of water within the well	2 (3%)	6.4	0.8–37.9
Well diameter (mm)	Diameter of the well	1 (1.5%)	143	51–1200
Casing material	Material of the well casing	4 (6%)	Steel: 34 wells PVC: 16 wells Concrete: 11 wells	NA
Conductivity (uS/cm)	Specific conductance spot measurement	7 (11%)	138	1.2–1014
Water temperature (°C)	Water temperature spot measurement	7 (11%)	13.2	9–15.3
pH	Scale of water acidity or basicity (0–14) spot measurement	7 (11%)	6.8	5.3–11.8
Dissolved oxygen (ppM)	Amount of dissolved oxygen spot measurement	7 (11%)	4.5	0.4–8.6
DOC (g/m^3^)	Dissolved organic carbon spot measurement	15 (24%)	2.1	0.2–23.6
DRP (mg/ m^3^)	Dissolved reactive phosphorus spot measurement	14 (23%)	0.003	0.001–0.06
NH4‐N (mg/ m^3^)	Ammoniacal nitrogen spot measurement	14 (23%)	11.5	2.0–774
NO3‐N (mg/ m^3^)	Nitrate nitrogen spot measurement	14 (23%)	1900	1.0–11000
TDN (mg/ m^3^)	Total dissolved nitrogen spot measurement	14 (23%)	2.04	0.02–10.9
TDP (mg/ m^3^)	Total dissolved phosphorus spot measurement	14 (23%)	0.003	0.001–0.06

## APPENDIX 2

The following keys are available and were used to morphologically identify amphipods and isopods:

Scarsbrook, M. R., G. D. Fenwick, I. C. Duggan, and M. Haase. 2003. A guide to the groundwater invertebrates of New Zealand. ISSN 1173–0382.

Fenwick, G.D. 2007. Quick guide to New Zealand freshwater amphipods. http://www.niwa.co.nz/sites/niwa.co.nz/files/amphipoda.pdf

Fenwick, G.D.; John, A. 2007. Quick guide to New Zealand freshwater Isopoda.

Fenwick, G.D.; Wilson, G.D.F. 2007. Field guide to New Zealand phreatoicid isopods. *NIWA Biodiversity Report*. 24 pp.

## APPENDIX 3

Sampling locations used in this study including North or South Island, region, aquifer and site (well) code, latitude, longitude, elevation, well depth, well casing type, sampling date, and availability of usable COI sequences obtained from the site (Yes/No)

**Table:**

Island	Region	Aquifer	Site	Latitude	Longitude	Elev (m)	Depth	Casing	Date	COI
North	Hawkes Bay	Ruataniwha	RU1	−39.9812	176.3265	250	23.5	Steel	18/10/2017	N
			RU2	−40.0082	176.3237	250	11.9	Steel	18/10/2017	N
			RU3	−39.8097	176.4742	232	7.7	NA	18/10/2017	N
			RU4	−39.8503	176.4525	215	21.2	Steel	18/10/2017	N
			RU5	−39.9019	176.4575	185	8.9	PVC	18/10/2017	N
			RU6	−39.9025	176.5147	160	6.3	Steel	19/10/2017	Y
			RU7	−39.9736	176.4914	150	6.9	Steel	17/10/2017	N
			RU8	−39.9654	176.644	110	38.7	Steel	19/10/2017	N
			RU9	−39.9653	176.644	110	7.9	Steel	19/10/2017	N
South	Canterbury	Central Plains	CP1	−43.483	171.9519	250	15	Steel	4/10/2017	N
			CP2	−43.4851	171.9516	245	15	Steel	4/10/2017	N
			CP3	−43.5015	171.9838	216	2.7	Steel	4/10/2017	Y
			CP4	−43.5017	171.9836	216	7.7	Steel	4/10/2017	Y
			CP5	−43.5629	171.9834	169	11.9	Steel	4/10/2017	Y
			CP6	−43.6084	172.0977	117	7	Steel	12/05/2017	Y
			CP7	−43.6076	172.0982	116	6.8	Steel	12/05/2017	N
			CP8	−43.6201	172.3091	60	18.3	PVC	5/10/2017	N
			CP9	−43.6202	172.3094	60	16.3	PVC	5/10/2017	N
			CP10	−43.6746	172.3217	35	6.5	Steel	12/05/2017	N
			CP11	−43.6743	172.3218	35	5.9	Steel	12/05/2017	N
			CPA	−43.6203	172.309	57	17.2	PVC	9/06/2015	N
			CPB	−43.6471	172.2298	67	18.3	Steel	30/05/2017	Y
			CPC	−43.7054	172.3606	21	15	Steel	1/03/2018	Y
		Rangitata Levels	RL1	−44.006	171.2551	192	9.4	Steel	8/11/2017	Y
			RL2	−44.0482	171.306	143	9.8	Steel	11/12/2017	N
			RL3	−44.0576	171.267	141	8.6	Steel	29/11/2017	Y
			RL4	−44.072	171.3319	117	12	Steel	29/11/2017	Y
			RL5	−44.1265	171.2723	82	7.6	Steel	8/11/2017	N
			RL6	−44.1477	171.2988	67	8.2	Steel	29/11/2017	Y
			RL7	−44.1645	171.3915	42	13.4	Steel	11/12/2017	Y
			RL8	−44.1922	171.3294	37	14.4	Steel	8/11/2017	N
			RL9	−44.2241	171.4141	7	6.3	Steel	29/11/2017	N
		Waimakariri‐Ashley	WA1	−43.2284	172.2278	220	6.8	PVC	16/06/2017	N
			WA2	−43.2338	172.405	130	5.3	Steel	6/11/2017	N
			WA3	−43.2492	172.49	105	5.8	PVC	6/11/2017	Y
			WA4	−43.3129	172.4826	75	26	PVC	14/11/2017	Y
			WA5	−43.2985	172.6162	27	15.2	Steel	6/11/2017	Y
			WA6	−43.2768	172.6142	25	5.3	Steel	16/06/2017	Y
			WA7	−43.2712	172.6272	21	6.3	PVC	9/11/2017	N
			WA8	−43.283	172.6412	20	14.1	Steel	11/12/2017	Y
			WA9	−43.274	172.6284	19	21.8	Steel	9/11/2017	N
			WA10	−43.3126	172.4822	18	19	PVC	14/11/2017	N
			WA11	−43.4165	172.6536	4	23.9	Steel	9/10/2017	Y
			WA12	−43.3443	172.4099	106	30	Steel	14/11/2017	N
	Tasman	Motueka	MT1	−41.477	172.8371	210	6.9	Steel	23/05/2017	N
			MT2	−41.2515	172.8217	78	7.3	Concrete	24/05/2017	N
			MT3	−41.2531	172.8225	75	6.8	Concrete	24/05/2017	N
			MT4	−41.1049	172.9994	8	19	Steel	24/05/2017	Y
			MT5	−41.1051	172.9992	8	18	uPVC	24/05/2017	N
			MT6	−41.1049	172.9998	8	21.5	uPVC	24/05/2017	N
			MT7	−41.1042	172.9996	8	10	uPVC	24/05/2017	Y
		Waimea	WM1	−41.4546	172.9574	143	4	Concrete	23/05/2017	N
			WM2	−41.4499	172.9595	140	4.3	Concrete	23/05/2017	N
			WM3	−41.4533	172.9577	139	3.9	Concrete	23/05/2017	N
			WM4	−41.4502	172.9622	132	3.4	Concrete	23/05/2017	N
			WM5	−41.3817	173.0811	90	9.2	PVC	25/05/2017	Y
			WM6	−41.3819	173.0813	90	9.2	PVC	25/05/2017	Y
			WM7	−41.3823	173.0817	90	8.6	PVC	25/05/2017	Y
			WM8	−41.3574	173.098	24	5	Concrete	23/05/2017	N
			WM9	−41.3568	173.097	23	5	Concrete	23/05/2017	N
			WM10	−41.2885	173.1243	3	10	PVC	23/05/2017	N
			WM11	−41.292	173.1195	3	6.4	Concrete	23/05/2017	N
			WM12	−41.2998	173.1412	3	14	NA	23/05/2017	N
	Southland	Mataura	MA1	−46.2981	168.8129	34	15.5	NA	26/10/2017	Y
			MA2	−46.3469	168.7855	28	15	NA	24/10/2017	Y

## APPENDIX 4

List of taxa and associated molecular operational taxonomic units (MOTU) based on Barcode of Life Datasystems (BOLD) Barcode Index Numbers (BIN). Site codes and unique BOLD Process IDs are also provided for each specimen

**Table:**

Order	Family	Genus species	MOTU (BIN)	Site	BOLD ID
Amphipoda	Paracrangonyctidae	*Paracrangonyx* sp.	BOLD:ADL2540	WM6	NZGDW023‐18
					NZGDW028‐18
				WM7	NZGDW024‐18
					NZGDW036‐18
					NZGDW039‐18
			BOLD:ADL3783	MT7	NZGDW029‐18
					NZGDW038‐18
					NZGDW040‐18
			BOLD:ADL5568	WA5	NZGDW079‐18
					NZGDW083‐18
	Paraleptamphopidae	*Paraleptamphopus* sp.	BOLD:AAB2704	CP3	NZGDW308‐18
					NZGDW311‐18
					NZGDW314‐18
					NZGDW315‐18
					NZGDW322‐18
					NZGDW324‐18
				CP5	NZGDW052‐18
					NZGDW068‐18
					NZGDW380‐18
				RL1	NZGDW001‐18
					NZGDW002‐18
					NZGDW013‐18
					NZGDW017‐18
				RL6	NZGDW215‐18
				WA8	NZGDW351‐18
		*Ringanui* sp.	BOLD:ADL2688	RL1	NZGDW008‐18
				RL3	NZGDW362‐18
			BOLD:ADL5144	WM6	NZGDW033‐18
			BOLD:ADL5178	WM6	NZGDW032‐18
					NZGDW035‐18
		Indeterminate	BOLD:ADL3023	WM7	NZGDW031‐18
			BOLD:ADL3150	WM6	NZGDW044‐18
					NZGDW057‐18
					NZGDW063‐18
					NZGDW067‐18
					NZGDW070‐18
				WM7	NZGDW043‐18
					NZGDW046‐18
					NZGDW047‐18
					NZGDW054‐18
			BOLD:ADL3507	MT7	NZGDW045‐18
					NZGDW050‐18
			BOLD:ADL3923	WA11	NZGDW003‐18
					NZGDW004‐18
					NZGDW010‐18
					NZGDW011‐18
					NZGDW012‐18
					NZGDW018‐18
					NZGDW089‐18
				WA3	NZGDW074‐18
				WA5	NZGDW075‐18
					NZGDW088‐18
			BOLD:ADL5343	CP4	NZGDW301‐18
					NZGDW303‐18
				CP5	NZGDW065‐18
			BOLD:ADO8356	CP3	NZGDW319‐18
					NZGDW331‐18
			BOLD:ADO8751	CP3	NZGDW313‐18
					NZGDW317‐18
				CP5	NZGDW058‐18
					NZGDW061‐18
					NZGDW066‐18
					NZGDW212‐18
					NZGDW294‐18
				CPC	NZGDW363‐18
				WA3	NZGDW073‐18
					NZGDW090‐18
				WA4	NZGDW198‐18
					NZGDW206‐18
					NZGDW208‐18
					NZGDW209‐18
					NZGDW217‐18
				WA5	NZGDW078‐18
					NZGDW080‐18
					NZGDW082‐18
					NZGDW087‐18
			BOLD:ADO9995	RL4	NZGDW199‐18
					NZGDW202‐18
					NZGDW210‐18
					NZGDW369‐18
			BOLD:ADP0945	CP4	NZGDW307‐18
			BOLD:ADP2782	CP3	NZGDW291‐18
					NZGDW305‐18
					NZGDW312‐18
					NZGDW316‐18
					NZGDW323‐18
					NZGDW325‐18
					NZGDW328‐18
					NZGDW329‐18
				CP4	NZGDW296‐18
				CP5	NZGDW216‐18
					NZGDW286‐18
					NZGDW297‐18
					NZGDW300‐18
				WA6	NZGDW337‐18
			BOLD:ADR4302	WM6	NZGDW041‐18
				WM7	NZGDW042‐18
				WM7	NZGDW049‐18
			BOLD:ADR9818	CP4	NZGDW288‐18
				WA3	NZGDW072‐18
					NZGDW081‐18
					NZGDW086‐18
			BOLD:ADS1859	MA1	NZGDW357‐18
					NZGDW358‐18
					NZGDW359‐18
				MA2	NZGDW356‐18
	Phreatogammaridae	Indeterminate	BOLD:AAC7108	CPB	NZGDW355‐18
			BOLD:AAC7110	RL1	NZGDW016‐18
			BOLD:ADL2601	MT7	NZGDW048‐18
			BOLD:ADL2933	CPC	NZGDW361‐18
				RL7	NZGDW338‐18
					NZGDW353‐18
				WA3	NZGDW085‐18
			BOLD:ADL3480	WM7	NZGDW053‐18
					NZGDW064‐18
					NZGDW069‐18
	Indeterminate	Indeterminate	BOLD:ADL2934	WA5	NZGDW076‐18
			BOLD:ADL3262	RU6	NZGDW077‐18
			BOLD:ADL3398	MT7	NZGDW060‐18
			BOLD:ADL5342	WM6	NZGDW059‐18
			BOLD:ADO7909	CP5	NZGDW302‐18
			BOLD:ADO7917	CP3	NZGDW320‐18
				CP4	NZGDW287‐18
					NZGDW293‐18
			BOLD:ADP0919	WM5	NZGDW341‐18
			BOLD:ADP1876	WA6	NZGDW333‐18
			BOLD:ADR2136	WM6	NZGDW056‐18
			BOLD:ADS6423	CP3	NZGDW330‐18
			BOLD:ADV1824	RL3	NZGDW332‐18
Isopoda	Janiridae	Indeterminate	BOLD:ADP3141	WA6	NZGDW342‐18
	Paranthuridae	*Cruregens fontanus*	BOLD:ADL2602	RL1	NZGDW006‐18
					NZGDW009‐18
				RL6	NZGDW200‐18
			BOLD:ADL3149	WM6	NZGDW022‐18
					NZGDW030‐18
					NZGDW034‐18
					NZGDW037‐18
				WM7	NZGDW027‐18
			BOLD:ADL3492	CP5	NZGDW051‐18
					NZGDW367‐18
					NZGDW371‐18
					NZGDW374‐18
					NZGDW376‐18
				WA8	NZGDW345‐18
					NZGDW349‐18
			BOLD:ADP0923	MT4	NZGDW348‐18
			BOLD:ADP4594	CP3	NZGDW310‐18
				CP6	NZGDW204‐18
	Tylidae	*Phreatoicus orarii*	BOLD:ADO9117	CP5	NZGDW194‐18
					NZGDW197‐18
					NZGDW379‐18
			BOLD:ADP4302	CP5	NZGDW195‐18
		*Phreatoicus typicus*	BOLD:ADP4667	CP4	NZGDW292‐18
					NZGDW304‐18
